# Rare Complication of Pneumomediastinum and Pneumopericardium in a Patient with COVID-19 Pneumonia

**DOI:** 10.1155/2020/8845256

**Published:** 2020-11-01

**Authors:** Anshika Singh, Jessica Bass, David H. Lindner

**Affiliations:** ^1^Internal Medicine PGY-2, NCH Healthcare System, Naples, FL 34102, USA; ^2^Internal Medicine PGY-3, NCH Healthcare System, Naples, FL 34102, USA; ^3^Chair, Pulmonary and Critical Care, Instructor of Medicine, Mayo School of Medicine, Associate Professor of Medicine, University of Central Florida, NCH Healthcare System, USA

## Abstract

Severe acute respiratory syndrome coronavirus 2 (SARS-CoV-2) is a recently discovered coronavirus which has caused a global outbreak of severe pneumonia with complications leading to hypoxic respiratory failure, acute respiratory distress syndrome (ARDS), cytokine storm, disseminated intravascular coagulation (DIC), and even gastrointestinal symptoms. While ground-glass opacity (GGO) is a typical radiographic finding associated most frequently with COVID-19 pneumonia, other less commonly noted atypical radiographic lung features include isolated lobar or segmental consolidation without GGO, discrete small nodules (centrilobular, “tree-in-bud”), lung cavitation, and smooth interlobular septal thickening with pleural effusion. Pneumomediastinum in COVID-19 patients has rarely been reported. A finding of pneumopericardium is unusual too. This report discusses the case of a young male with COVID-19 pneumonia who was found to have both these features on computed tomographic (CT) scans of his chest on presentation.

## 1. Introduction

As we navigate the current global public health emergency due to the COVID-19 pandemic, new and atypical clinical manifestations of this disease continue to emerge. Several studies analyzing the complications and clinical outcomes in the confirmed cases of SARS-CoV-2 have shown respiratory failure, ARDS, cytokine storm, DIC, and even gastrointestinal symptoms as the dominant feature in patients with moderate to severe disease. Typical radiographic findings are multifocal ground-glass opacities on computed tomography (CT) scans of the chest [1]. We present an unusual case of a young male patient with COVID-19 pneumonia who was found to have pneumomediastinum and pneumopericardium on imaging. On an extensive literature search, so far, about four such cases have been reported where pneumomediastinum was deemed as a probable rare complication of COVID-19 pneumonia [2]. To the best of our knowledge, no case with pneumopericardium has been reported thus far. The mechanism of developing this probable complication in COVID-19 pneumonia is poorly understood and our report attempts to explore this. The implications of these findings in terms of indicators of disease severity and impact on clinical outcome open an important scope for further discussion.

## 2. Case Summary

**Figure 1:**
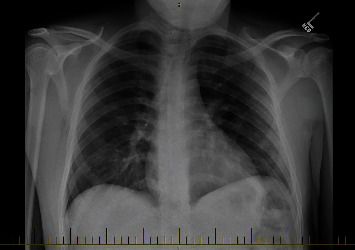
Faint patchy infiltrates of both lungs noted.

**Figure 2:**
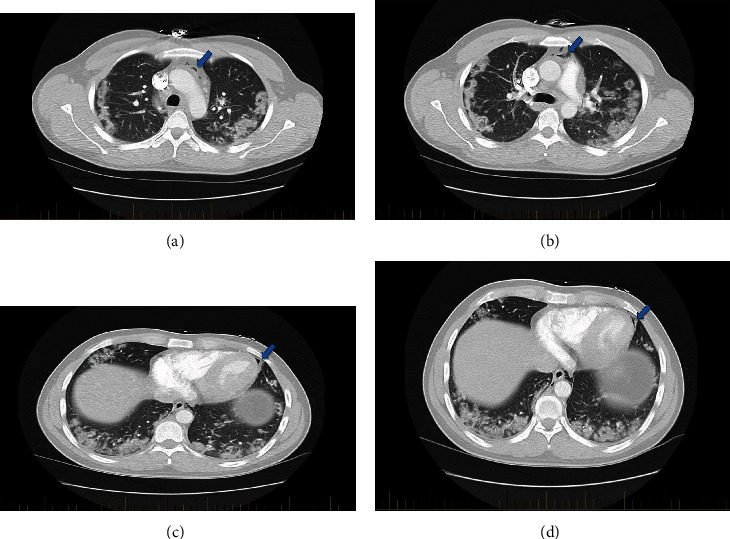
(a) Computed tomographic scans of the chest demonstrating bilateral peripheral ground-glass opacities along with pneumomediastinum (blue arrow). (b) Computed tomographic scans of the chest demonstrating bilateral peripheral ground-glass opacities along with pneumomediastinum (blue arrow). (c) Computed tomographic scans of the chest demonstrating bilateral peripheral ground-glass opacities along with pneumopericardium (blue arrow). (d) Computed tomographic scans of the chest demonstrating bilateral peripheral ground-glass opacities along with pneumopericardium (blue arrow).

A 33-year-old Hispanic male with no known chronic health issues presented to the emergency department with complaints of subjective fever, body aches, worsening shortness of breath, cough, and headache for 13 days. He also complained of sharp retrosternal chest pain. He denied exposure to known COVID-19 positive contacts and reported no known allergies, recent travel, or active medications. Social history was consistent with occasional alcohol consumption, lifelong nonsmoker, and no history of illicit substance abuse. He did not recall any significant family history. On presentation, his oral temperature was 39.5 C, heart rate 80/min, respiratory rate 26/min with an oxygen saturation of 96% on room air, and blood pressure 113/74 mmHg. Physical examination was notable for tachypnea with clear lungs on auscultation and no visible signs of respiratory distress. Heart auscultation demonstrated no identifiable rubs, murmur, or bruits on cardiovascular examination. Laboratory findings including complete blood count, basic metabolic panel, and inflammatory markers were as follows (Table 1). He was positive for SARS-CoV-2 based on RT-PCR analysis of nasopharyngeal swab. Arterial blood gas on room air showed hypoxia with pO2 68, pCO2 42, and pH 7.4. Faint patchy infiltrates in both lungs were seen on a portable chest X-ray (Figure 1). On chest CT, multiple confluent areas of ground-glass opacification and alveolar consolidation were present throughout the peripheries of both lungs. There was no evidence of pulmonary embolism; however, pneumomediastinum and pneumopericardium were noted (Figures 2(a)–2(d)).

Our patient had a hospital course (with appropriate droplet and contact precautions) of six days requiring 3 L supplemental oxygen via nasal cannula to maintain oxygen saturation between 88-92%. We avoided positive pressure ventilation to prevent the alveoli from further barotrauma. He received oral azithromycin for five days, and in the absence of severely elevated inflammatory markers and cytokine storm, the need for dexamethasone or tocilizumab was not felt. In the absence of a more severe disease, we did not consider him a suitable candidate for Remdesivir. He was weaned off the supplemental oxygen successfully. Shortness of breath, chest pain, and cough were resolved on day 3. His temperature on the day of discharge was 36.8 C, heart rate 63/min and respiratory rate 16/min, and was saturating at O2 94% on room air. Repeat chest CT showed decreasing pneumomediastinum and persistent bilateral consolidation. He was discharged home with advice to follow-up with the pulmonology clinic in the outpatient setting.

## 3. Discussion

Pneumomediastinum is defined as free air in the mediastinum and is broadly classified as spontaneous pneumomediastinum (SPM) and secondary pneumomediastinum. SPM is usually benign and idiopathic while secondary pneumomediastinum results from trauma, intrathoracic infections, esophageal rupture, or tears along the tracheobronchial tree [3]. As seen in our patient, most cases of pneumomediastinum present with acute retrosternal chest pain often worsened by inspiratory maneuvers. The Macklin effect is a generally accepted mechanism for the development of SPM, which proposes that free air from ruptured alveoli tracks back along the bronchovascular sheath and into the mediastinum. Air present in the mediastinum can further dissect through the periaortic tissue plane and cause pneumopericardium. Comorbidities often deemed as triggering factors include asthma exacerbation and Valsalva effect during labor, cough, and hyperemesis, though most of the cases remain idiopathic [3, 4]. Although there is some literature on cases of viral pneumonia complicated by pneumomediastinum due to violent coughing in paediatric patients with human bocavirus and H1N1, its incidence in adults with COVID-19 pneumonia remains rare and atypical [5]. Whether detection of pneumomediastinum in COVID-19 pneumonia should be considered as spontaneous or secondary to the virus is still debatable.

Our patient is a young male with a history negative for potential triggers for pneumomediastinum such as asthma, smoking, cocaine abuse, emphysema, hyperemesis, or external or iatrogenic trauma. The exact mechanism of pneumomediastinum is not fully understood in COVID-19 pneumonia. It is plausible that increased intrathoracic pressure from constant coughing in combination with decreased pressure in perialveolar interstitial space from increased respiratory efforts led to alveolar rupture and back-tracking of free air as theorized by the Macklin effect. Chest CT scan has emerged as the gold standard for diagnosing pneumomediastinum. Typical chest CT findings most commonly associated with COVID-19 pneumonia are reported as peripheral, multifocal, bilateral, ground-glass opacity (GGO) with or without consolidation, or visible intralobular lines. Atypical findings are characterized by the absence of above typical features and presence of isolated lobar or segmental consolidation without GGO, discrete small nodules (centrilobular, “tree-in-bud”), lung cavitation, and smooth interlobular septal thickening with pleural effusion [6]. In addition to our patient, we were able to find reports on three other RT-PCR confirmed COVID-19 cases with radiographic evidence of pneumomediastinum [7–9], making this another new atypical finding worth keeping in mind while reading and reviewing the chest images of these patients. While SPM is generally a benign phenomenon, we believe that early detection would help clinicians be vigilant of any potential respiratory and circulatory collapse that can accompany secondary pneumomediastinum [3, 10].

Notably, the peculiar finding of pneumopericardium in COVID-19 pneumonia has yet to be reported in the literature. Pneumopericardium is characterized by the presence of air in the pericardial cavity that can result from blunt chest wall trauma in deceleration injury and as a rare complication of pericardiocentesis or positive pressure ventilation. The pericardium is a sac covering the heart that joins the vascular sheath of large blood vessels and the hilum of the lung. It is possible that at higher intrathoracic pressure, the air from ruptured alveoli can dissect through the superior and anterior mediastinal part and hilum opening of the lungs and enter the pericardium along venous sheaths which have lower collagenous support [11]. Although this makes anatomical sense, we cannot say with absolute certainty that the same explanation can be applied in our COVID-19 patient who presented with both pneumomediastinum and pneumopericardium. Most adult patients with pneumopericardium remain asymptomatic but can sometimes develop the complication of tension pneumopericardium leading to hemodynamic instability [12]. Since COVID-19 patients are at high risk for respiratory failure, we suggest even apparently benign cases of pneumopericardium should be closely monitored. Although it is not uncommon for pneumomediastinum to be a self-limited process, it is still early to determine its implication on disease severity and clinical outcome when in association with COVID-19 pneumonia. Recurrent pneumomediastinum remains an even rarer phenomenon, with just about five reported cases [3]. It would be interesting to monitor if recovered COVID-19 patients develop recurrent pneumomediastinum later in their lives.

## Figures and Tables

**Table 1:** 

Significant labs	On presentation	On day of discharge	Reference range
Leukocytes	3.9 th/ul	5.2 th/ul	4.2-10.8 th/ul
Red blood cells	4.69 mil/ul	4.31 mil/ul	4-5.40 mil/ul
Hemoglobin	14.5 gm/dL	13.1 gm/dL	14-18 gm/dl
Platelets	132 th/ul	426 th/ul	130-450 th/ul
Granulocytes	79.6%	61.1%	41-77%
Lymphocytes	14.5%	22.5%	24-44%
Monocytes	5.3%	11.3%	0-15%
Eosinophils	3.3%	3.2%	0-5.0%
Basophils	0.3%	0.8%	0-3.0%
Neutrophil absolute	3.14 th/ul	3.2 th/ul	1.8-7.0%
Potassium	3.8 mmol/L	4.3 mmol/L	3.5-5.1 mmol/L
Sodium	135 mmol/L	137 mmol/L	136-145 mmol/L
Chloride	102 mmol/L	104 mmol/L	98-107 mmol/L
Bicarb	25 mmol/L	30 mmol/L	21-32 mmol/L
BUN	13 mg/dL	12 mg/dL	7-18 mg/dL
Creatinine	1.1 mg/dL	0.8 mg/dL	0.6-1.3 mg/dL
Erythrocyte sedimentation rate	91 mm/hr	Not rechecked	0-10 mm/hr
Lactate dehydrogenase	325 IU/L	305 IU/L	87-241 IU/L
C reactive protein (CRP)	16.4 mg/dL	1.5 mg/dL	<0.03 mg/dL
Procalcitonin	0.11 ng/ml	0.05 ng/ml	<0.05 ng/ml
Ferritin	910 ng/ml	849 ng/ml	26-388 ng/ml
D-dimer	1.96 ug/ml	0.43 ug/ml	0-0.06 ug/ml

## References

[1] Huang C., Wang Y., Li X. (2020). Clinical features of patients infected with 2019 novel coronavirus in Wuhan, China. *The Lancet*.

[2] Mohan V., Tauseen R. A. (2020). Spontaneous pneumomediastinum in COVID-19. *BMJ Case Reports*.

[3] Caceres M., Ali S. Z., Braud R., Weiman D., Garrett H. E. (2008). Spontaneous pneumomediastinum: a comparative study and review of the literature. *The Annals of Thoracic Surgery*.

[4] Wintermark M., Schnyder P. (2001). The Macklin effect. *Chest*.

[5] Emiralioğlu N., Ozcan H. N., Oğuz B. (2015). Pneumomediastinum, pneumorrhachis and subcutaneous emphysema associated with viral infections: report of three cases. *Pediatrics International*.

[6] Simpson S., Kay F. U., Abbara S. (2020). Radiological Society of North America Expert Consensus Statement on reporting chest CT findings related to COVID-19. Endorsed by the Society of Thoracic Radiology, the American College of Radiology, and RSNA. *Radiology: Cardiothoracic Imaging*.

[7] Wang J., Su X., Zhang T., Zheng C. (2020). Spontaneous pneumomediastinum: a probable unusual complication of coronavirus disease 2019 (COVID-19) pneumonia. *Korean Journal of Radiology*.

[8] Sun R., Liu H., Wang X. (2020). Mediastinal emphysema, giant bulla, and pneumothorax developed during the course of COVID-19 pneumonia. *Korean Journal of Radiology*.

[9] Zhou C., Gao C., Xie Y., Xu M. (2020). COVID-19 with spontaneous pneumomediastinum. *The Lancet Infectious Diseases*.

[10] Kouritas V. K., Papagiannopoulos K., Lazaridis G. (2015). Pneumomediastinum. *Journal of Thoracic Disease*.

[11] Lee H. U., Kang D., Lee J. C. (2018). Pneumomediastinum and pneumopericardium as rare complications after retroperitoneal transpsoas lateral lumbar interbody fusion surgery: a case report. *Medicine*.

[12] Lee J., Kang B. S., Kim C., Choi H. J. (2016). Tension pneumopericardium after pericardiocentesis. *Journal of Korean Medical Science*.

